# Supplementary material for: A UMLS-based spell checker for natural language processing in vaccine safety
Source: BMC Med Inform Decis Mak. 2007 Feb 12;7:3. doi: 10.1186/1472-6947-7-3 (PMC1805499; doi:10.1186/1472-6947-7-3)
Supplement: Additional file 13 — RAP application directory. Sets up directory for RAP (RDF application for PHP) source code [file 1472-6947-7-3-S13.gz › rap/doc/phpdoc/__filesource/fsource_default__vocabularyRSS.php.html]

xml version="1.0" encoding="iso-8859-1"?


File Source for RSS.php


# Source for file RSS.php

Documentation is available at RSS.php

```
1. <?PHP
2. // ----------------------------------------------------------------------------------
3. // RSS Vocabulary
4. // ----------------------------------------------------------------------------------
5. // Version                   : 0.9
6. // Authors                   : Tobias Gau� (tobias.gauss@web.de)
7. //
8. // Description               : Wrapper, defining resources for all terms of RSS.
9. //                               For details about RSS see: http://purl.org/rss/1.0/.
10. //                                Using the wrapper allows you to define all aspects of
11. //                             the vocabulary in one spot, simplifing implementation and
12. //                             maintainence. Working with the vocabulary, you should use
13. //                             these resources as shortcuts in your code.
14. //
15. // ----------------------------------------------------------------------------------
16. // History:
17. // 11-08-2003                 : Initial version
18. // ----------------------------------------------------------------------------------

20. // RSS concepts

22. $RSS_channel = new Resource(RSS_NS . 'channel');
23. $RSS_image = new Resource(RSS_NS . 'image');
24. $RSS_item = new Resource(RSS_NS . 'item');
25. $RSS_textinput = new Resource(RSS_NS . 'textinput');

27. $RSS_items = new Resource(RSS_NS . 'items');
28. $RSS_title = new Resource(RSS_NS . 'title');
29. $RSS_link = new Resource(RSS_NS . 'link');
30. $RSS_url = new Resource(RSS_NS . 'url');
31. $RSS_description = new Resource(RSS_NS . 'description');
32. $RSS_name = new Resource(RSS_NS . 'name');


39. ?>
```

Documentation generated on Fri, 17 Dec 2004 16:18:06 +0100 by phpDocumentor 1.3.0RC3
